# Enchondroma versus Chondrosarcoma in Long Bones of Appendicular Skeleton: Clinical and Radiological Criteria—A Follow-Up

**DOI:** 10.1155/2016/8262079

**Published:** 2016-02-23

**Authors:** Eugenio M. Ferrer-Santacreu, Eduardo J. Ortiz-Cruz, Mariana Díaz-Almirón, Jose Juan Pozo Kreilinger

**Affiliations:** ^1^Orthopaedic Surgery Department, Hospital Universitario de Móstoles, C/Río Júcar s/n, 28935 Móstoles, Spain; ^2^Orthopaedic Oncology Unit, Orthopaedic Surgery Department, Hospital Universitario La Paz, Paseo de la Castellana 261, 28046 Madrid, Spain; ^3^Hospital La Paz Research Institute (IdiPaz), Paseo de la Castellana 261, 28046 Madrid, Spain; ^4^Pathology Department, Hospital Universitario La Paz, Paseo de la Castellana 261, 28046 Madrid, Spain

## Abstract

As of today two types of cartilage tumors remain a challenge even for the orthopedic oncologist: enchondroma (E), a benign tumor, and chondrosarcoma (LGC), a malignant and low aggressiveness tumor. A prospective study of 133 patients with a cartilaginous tumor of low aggressiveness in the long bones of the appendicular skeleton was done to prove this difficult differential diagnosis. Parameters including medical history and radiological and nuclear imaging were collected and compared to the result of the biopsy. A scale of aggressiveness was applied to each patient according to the number of aggressiveness episodes present. A comparison of the results of the biopsy with the initial diagnosis made by the orthopedic oncologist based solely on clinical data and imaging tests was also made. Finally, a management algorithm for these cases was proposed. A statistical significance for LGC resulted from the parameter as follows: pain on palpation, involvement of cortical in either the CT or MRI, and Tc99 bone scan uptake equal or superior to anterosuperior iliac crest. In our series, a tumor scoring 5 points or higher in the scale of aggressiveness can have 50% more chance of being LGC. When compared with the gold standard (the biopsy), surgeon's initial judgement showed a sensitivity of 73.5% and a specificity of 94.1%.

## 1. Introduction

Distinction between enchondroma (E) and low-grade chondrosarcoma (LGC) remains a challenge for any specialist on musculoskeletal sarcomas management including orthopaedic surgeons, pathologists, and radiologists (Table 1). Even in the most expert hands, these two entities can lead to a wrong diagnosis and, as a consequence, to an unsuitable treatment [1–3]. No previous published study has been able to show any distinctive feature between E and LGC in long bones of the appendicular skeleton [4–7]. An initial diagnosis based upon clinical, radiological, and metabolical data is capital, because the biopsy does not provide always an accurate result.

Firstly, our aim with this study was to find out if there was any feature enabling us to differentiate between E and LGC without performing any invasive procedure, as biopsy. On that purpose, we performed a prospective data collection of patients having a low aggressiveness chondral tumor in long bones of appendicular skeleton including information from their clinical stories and imaging. We also included the initial diagnosis based upon clinical and imaging data made by a single experienced sarcoma surgeon. Correlation between the biopsy and each radiological and clinical feature, as well as surgeon's initial diagnosis, was performed. Secondly, we elaborated an aggressiveness score which could serve as a tool in decision-making based on clinical, radiological, and metabolical features. Finally, we have made a management algorithm proposal based on our findings for the management of these patients.

## 2. Patients and Methods

### 2.1. Data Collection

We have performed a prospective study in which 182 patients presenting a low aggressiveness cartilage-type lesion on plain radiographs in long bones of appendicular skeleton suggestive of E or LGC have been included. In the first visit to the clinic, personal and clinical data were collected. Further imaging (CT, MRI, and bone scan) including reports done by specialists were carefully collected to complete our database. We also included specialist's first diagnostic impression based upon clinical and imaging data and the final result of the biopsy. Patients under 18 years old, cartilage lesions in hands, feet, and axial skeleton, cases of enchondromatosis (including Ollier's disease and Maffucci's syndrome), osteochondromatosis, secondary chondrosarcomas or chondrosarcomas of intermediate or high grade according to Evans classification, and recurrences of previously operated tumors were excluded.

In each patient, a form was filled with personal data, physical examination, and symptoms (focusing on pain and its features: presence of pain with palpation, inflammatory or mechanic, evolution, etc.). Concerning age, patients were divided into two groups: up to 35 years of age and more than 35 years of age. The site of the tumor (bone, side, and bone area) was registered. In plain radiographs, we measured size, site, and appearance and changes in calcification over time. In CT, size, calcification (presence and changes over time) endosteal scalloping, and soft tissue mass (STM) were registered. In MRI, size, endosteal scalloping, and STM were also recorded. Concerning Tc99 bone scan, lesions were classified according to the presence of radionuclide uptake on whole-body image. The degree of uptake was compared to the physiological uptake of the iliac crest (similar to or lower or higher than iliac crest uptake) focusing on the anterosuperior iliac crest (ASIC), as recorded by nuclear medicine specialists in their reports. In each case, an initial diagnosis (E or LGC) was made by a single specialist in musculoskeletal oncology surgery based upon clinical, radiological, and metabolic data. A decision of performing a biopsy or just doing a follow-up of the patient was made after this initial diagnosis. A record of the biopsy and final result was also included and all specimens were reviewed by the same department. The judgment made by the pathologist could confirm or reject surgeon's initial impression. In those cases in which an E was suspected, patients were followed periodically. If, after three years of follow-up, no changes in clinical or radiological features were registered, those cases were assumed hypothetically as E although diagnosis was made only based upon clinical and radiological criteria.

### 2.2. Aggressiveness Scale

As part of each patient's evaluation, an account of features indicating aggressiveness was performed (Table 2). One point was given to every feature of aggressiveness shown by the lesion in three categories: clinical (CA), radiological (RA), and metabolic (MA). As well, a final score was obtained with the sum of the score obtained in each category. Statistical significance between biopsy's result and the score obtained in each category as well as the final score was calculated.

### 2.3. Statistical Analysis

Statistical analysis of the collected data was performed by Hospital La Paz University Research Institute (IdIPaz). All statistical tests were bilateral and significance was considered when *P* values were under 0.05. Software employed was SAS 9.1 (SAS Institute Inc, Cary, NC, USA).

Quantitative data description of our series consisted of mean and standard deviation and median, minimal, and maximal values. To evaluate the accuracy of the first diagnosis made by the surgeon compared to the gold standard (biopsy's result) in each case, sensitivity and specificity, as well as false positive and false negative rates, were calculated. In order to establish statistical relationship between every feature included in the study and the possibility of being an E or a LGC, *P* values obtained by means of the Fisher's exact test and chi-square test were considered. This was also calculated between the scores obtained in every aggressiveness category.

As well, relationship between total score (TS) in the aggressiveness scale (AS) and biopsy's result was calculated. A statistical model was developed to show the risk increase of having a LGC with every additional point, that is, with every additional feature of aggressiveness shown by the lesion.

### 2.4. Literature Review

Previous peer-reviewed literature on the matter has been revised with the aid of Pubmed and Ovid databases using the following keywords: “enchondroma versus low grade chondrosarcoma” and “chondral tumors diagnosis”. Papers older than 20 years were discarded unless they were considered as classics by experts.

### 2.5. Ethical Issues

According to our country legal requirements, patients were informed and gave a verbal consent to allow us to use their clinical data in this research. As well, we obtained a certificate of approval from the ethical committee for clinical research in our institution.

## 3. Results

### 3.1. Clinical, Radiological, and Metabolic Features

A prospective study has been performed in which 182 patients were included. Twenty-two variables were registered for analysis. At the end, only 133 patients completed the follow-up. Of these, 39 were diagnosed as E (29.3%) and 94 as LGC (70.7%).

A biopsy was performed in 90 patients (13 were percutaneous and CT-guided, 9 were incisional, and 68 were excisional). The remaining 43 patients were followed up because they had a chondral tumor without any sign of clinical or radiological aggressiveness. As explained above in the patients and methods section, they were considered as E.

Our series consisted of 33 men (24.8%) with a mean age of 49.8 years and 100 women with a mean age of 49.8 years. Global mean age in our series was 50.1 years. In patients finally diagnosed as E, 25.3% were male and 74.7% were female. In those having a LGC, 23.5% were male and 76.5% female. Regardless of these differences in gender distribution, statistical analysis showed no relevance (*P* = 0.494). Concerning age groups, 14.3% of the patients were less than 35 years of age and 82.7% were of that age or older. Among those diagnosed as E, 16.7% were under 35 years of age and 83.3% were older. In the LGC group, 11.8% were less than 35 years of age and 88.2% were older. Analysis showed no relevance between final diagnosis and belonging to one specific age group (*P* = 0.307). Most common bone affected was the femur (44.1%) followed by the humerus (37.5%). Fibula (8.82%) and tibia (8.2%) were not so frequent in our study (Table 3). These differences turned out to be irrelevant when analyzed (*P* = 0.575). Among cases of E, 55.7% were on the right side and 44.3% on the left. In the LGC group, 39.2% of the lesions were on the right side while 60.8% were on the left. No relevance was found regarding this matter (*P* = 0.116).

Concerning the most frequent bone site affected, proximal metaphysis (36.09% of the cases) and distal metaphyso-epiphyseal zone (24.81%) followed by proximal epiphysometaphyseal zone (17.29%) were the three top sites in our study.  Table 4 shows locations distribution by diagnosis. When analyzed, these data showed no statistical relationship with final diagnosis (*P* = 0.575).

Almost 60% of the cases were casual findings when the involved area was studied for other reasons, mostly pain or traumatism. In 61.2% of the patients pain had a mechanical pattern, while in 29.5% of the patients, pain was inflammatory. 9.3% of the patients had no pain at all. In the E group, 14.1% were asymptomatic whereas only 2% of the LGC showed no symptoms at all. Among those patients with E who had pain, in 74.6% pain was mechanic (58% in the LGC) and in 25.4% pain was inflammatory (42% in the LGC). Analyzing these data, *P* value was 0.111. No statistical significance was found for this feature. In the physical examination, 70.67% of the patients had pain with palpation. Among patients with E, 58.2% had pain with palpation and among patients with LGC, proportion reached 88.2%. Statistical analysis showed *P* < 0.001 establishing statistical relevance between the presence of pain during physical examination and the possibility of the lesion being a LGC.

Size measured on plain radiographs was bigger than 5 cm in 40.61% of the cases. Analysis sowed *P* = 0.326 with no relevance for these differences (Table 5).

There were no cases of soft tissue mass in CT or MRI in our study. Cortex involvement in CT imaging was seen in 63.9% of the cases. As explained in the patients and methods section, involvement of the cortical bone was divided into three categories, according to the depth affected by the tumor: one-third, two-thirds, or total involvement (Table 6). Analysis showed *P* < 0.01 because most cases without cortical involvement (36.09%) were finally diagnosed as E. A relationship between E and lack of cortical involvement was present in our study.

In MRI, 31.7% of our series showed some cortical involvement, which represents half the cases detected with CT scan.  Table 7 shows the MRI cortical involvement in each diagnosis. Statistical analysis showed *P* < 0.001 which is understandable after seeing that most E do not have cortical involvement and most LGC do. Because of this situation, patients were redistributed in two groups: cortical involvement in CT or MRI and no cortical involvement in CT and MRI.

Technetium 99 bone scan was positive in some degree in 97.5% of the patients in our study. 97.2% of the E and 97.5% of the LGC showed some uptake. As there was almost no difference between both groups, no significance was found after analysis (*P* = 0.652). Comparison between tumor's uptake and anterosuperior iliac crest (ASIC) physiological uptake is shown in Table 8. No statistical differences were detected between those cases having higher uptake and showing a similar uptake. But, if these two categories were considered as one and compared to those patients showing lower uptake than ASIC, then *P* < 0.01 which is statistically significant. Significance was also found in the fact that 82.8% of the cases showing lower uptake were finally diagnosed as E and 17.2% as LGC (*P* < 0.01).

To summarize our findings so far, pain with palpation, cortical involvement in any degree in CT or MRI, and a similar or higher Tc99 uptake than ASIC showed statistical relevance in the possibility of this lesion being a LGC.

### 3.2. Aggressiveness Score

As explained in the Patients and Methods, we employed an aggressiveness score in each patient giving one point for each feature of aggressiveness shown by the lesion in three fields: clinical (CA), radiological (RA), and metabolic (MA). Adding the points obtained in the three categories, we obtained a final score or total aggressiveness (TA). Considering CA, patients with E scored 0 points in 30.2% of the cases, 1 point in 57%, and 2 points in 12.7%. Among those having LGC, only 6% scored 0 points, 60% scored 1 point, and 34% scored two points. From another point of view, among patients having 0 points of CA, 88.9% were E and 11.1% were LGC. Among those patients scoring 1 point, 60% were E and 40% LGC. Finally, patients scoring 2 points turned out to be LGC in 63% of the cases and E in 37% of the cases. Analysis showed *P* < 0.01 for these differences, revealing a high possibility of E when CA is 0 and LGC when CA was 2.

Among patients having an E 3.8% scored 0 points (we had no cases among the LGC); 79.2% scored 1 point (20.8% of the LGC); 51.9% scored 2 points (48.1% of the LGC); 8.9% scored 3 points (28% of the LGC). Statistical analysis showed significance in the possibility of having an E when RA was 1 and having a LGC when RA was 3. Differences in the 0 points' group were not significant because only three patients were in that category (3 E and no LGC).

Finally, metabolic aggressiveness (MA) showed that 12.7% of the E had 0 points (8% of the LGC), 32.9% had 1 point (12% of the LGC), and 54.4% had 2 points (80% of the LGC). On the other hand, among those patients scoring 0 points, 71.4% were E and 28.6% LGC; among those scoring 1 point 81.2% were E and 18.8% LGC. Finally, among those scoring 2 points, 61.2% were E and 38.8% were LGC. These differences were statistically significant when analyzed (*P* < 0.01). When considering the final score or total aggressiveness, analysis showed that every new point increased the possibility of having a LGC rather than an E. Specifically, every new point in the score multiplies the risk of having a LGC by 2.3 (*P* < 0.01). Our model established that a patient scoring 5 points or more in the aggressiveness score had more than 50% of possibilities of having a LGC.

### 3.3. Expert's Initial Judgement

The surgeon in charge of the initial diagnosis classified 64 of the cases as LGC, of which 61 were confirmed by pathologists. On the other hand, 69 were classified as E but only 47 were confirmed as such and the other 22 patients were reclassified as LGC. Sensitivity in our series was 73.5% and specificity was 94.1% for diagnosis based exclusively on clinical and radiological features. Positive predictive value was 93.5% and negative predictive value was 68.1%. False positive rate was 5.9% while false negative rate was 26.5% (confidence interval of 95%).

## 4. Discussion

Distinction between clinical E and LGC remains a challenge for specialists in bone and soft tissue sarcomas. So far, we do not know a paper that has analyzed the relationship between specific clinical, radiological, and metabolic features and the possibility of that lesion to be an E or a LGC in long bones, except for the one published by this group in 2012 [4]. Our reference in the literature has been the study published by Murphey et al. [5, 6] in which this same idea was developed but taking into account chondrosarcomas of intermediate and high degree as well. They found relevant differences in the following features: gender, size, metaphyso-epiphyseal location for LGC and diaphyseal location for E, pain, STM, endosteal scalloping both in depth and in length, histological pattern, cortical remodeling, periosteal reaction, degree of mineralization, amount and homogeneity of Tc99 uptake in bone scan, pathologic fracture, and cortical thickening. In their study on the usefulness of radiographs and clinical data, Geirnaardt et al. [7] obtained relevant differences favoring LGC when the lesion was in axial skeleton and was bigger than 5 cm. Our previous paper in 2011 [4], also focused on long bones in appendicular skeleton and including 82 patients, showed no relevant differences between E and LGC when comparing the same clinical, radiological, and features analyzed in this paper. Our first hypothesis proposed the absence of any feature which had a statistical relationship with the final diagnosis of E or LGC in the biopsy. Statistical analysis in our series has shown that pain with palpation, cortical involvement in CT or MRI, and uptake in Tc99 bone scan similar or higher than ASIC had significant relationship with a final diagnosis of LGC. For that reason, we have rejected our initial hypothesis.

### 4.1. Clinical Findings and Plain Radiograph

In our study the only clinical feature showing statistical relationship with LGC has been pain with palpation. No differences were found concerning the type of pain or features found in the radiographs. Presence of pain, especially when having inflammatory pattern, has always been related to malignancy but in our series we did not find a relationship between this type of pain and LGC. Murphey et al. [5], in their study, found that tumor's size, pain of any kind, patient's age and gender, and tumor location (Metaphyso-epiphyseal for LGC and diaphyseal for E) were statistically significant to distinguish between E and LGC. We must remember that Murphey's study included chondrosarcomas of all grades. Geirnaardt et al. [7] reached the conclusion in their study that a tumor bigger than five cm in a plain radiograph was significant for LGC (including tumors in axial skeleton). Nevertheless, they found differences for clinical symptoms and stressed the scarce usefulness of these features to reach a correct diagnosis. Nevertheless, we consider that the presence of pain with palpation or inflammatory pain should make the clinician perform further imaging studies for a more accurate decision-making.

### 4.2. Imaging Studies

Imaging includes CT scan, MRI scan, and Tc99 bone scan. Some studies have tried to analyze medullary perilesional edema or contrast enhancement but we did not include these features because they were not part of the standard management of a low aggressiveness cartilage-like tumor [8–10]. In our series, no STM has been found. Concerning cortical involvement, our initial classification in one-third, two-thirds, or complete involvement did not show any differences. However when we redistributed our patients in two categories, that is, cortical involvement or no cortical involvement in CT or MRI, we detected an association between cortical involvement of any degree and final diagnosis of LGC in both CT or MRI. Moreover, these differences were especially significant in MRI images. This finding is of special interest considering that CT scan is supposed to provide a better detection of cortical involvement than MRI. Our conclusion was that the better sensitivity of CT scan in this matter makes it possible that involvement can be found even if it is of very small degree. This makes it possible that E and LGC show almost no differences between them in CT scan imaging. On the other hand, MRI only detects cortical involvement when it has certain degree and considering that LGC usually is more aggressive in its growth, it is easier to find differences between LGC and E in MRI images. This fact, as it will be discussed later, led us to avoid CT in the standard imaging protocol of these tumors and keep it only for patients in which an MRI is not available for some reason. In Tc99 bone scan, uptake presence and comparison with ASIC's physiological uptake were analyzed. Murphey et al. detected differences between E and chondrosarcomas of all grades. They found that a higher degree of uptake and its uniformity was an indication of malignancy. In our series, most patients showed some degree of uptake. When comparing with ASIC's uptake, analysis showed that patients with similar or higher uptake had more possibilities of having a LGC. This feature was not considered in other studies but has been included in the most recent management algorithms.

### 4.3. Aggressiveness Score

Another goal in our study was to elaborate an aggressiveness score (AS) as a tool to measure the likelihood of a low aggressiveness cartilage tumor to be a LGC. It was a support in doubtful cases to complement clinical and radiological data and in no way can be considered as an absolute indicator of malignancy. So far, we have no notice of any score of this kind being published in peer-reviewed literature. We must make it clear that the AS should be used with tumors in long bones of appendicular skeleton where differential diagnosis between E and LGC is complicated. As explained before, in the material and method section, this score includes three categories of evaluation: clinical aggressiveness (CA) with a top score of two points, radiological aggressiveness (RA) with a top score of four points, and metabolic aggressiveness (MA) with a top score of two points. Finally, the sum of the three categories provides a total aggressiveness (TA) score. Statistical analysis of the scores obtained in our patients showed significant differences for each category separately and for the final score indicating that the higher the score is, the higher the possibility of that tumor to be a LGC. It is remarkable that each feature showing significance in the first part of our study belongs to one of the three different categories of the score, that is, pain with palpation to CA, cortical involvement in CT or MRI to RA, and bone scan uptake similar or higher than ASIC to MA. A prediction model was established to appreciate the risk increase with every additional point obtained in the AS for each patient. A critical score was also established for the score in which the risk of having a LGC was higher than having an E. Our analysis established that those patients with a TA score of 5 or more had a risk of having LGC higher than 50%. We must consider that this model does not establish whether these points come from clinical, radiological, or metabolic features.

### 4.4. Specialist's Initial Diagnosis versus Final Diagnosis

The expert's opinion in the present study has shown a sensitivity of 73.5% and a specificity of 94.1% compared to biopsy. As mentioned at the beginning of this study, it is of key importance to have an algorithm to increase the sensitivity so no patient with a LGC is considered as an E. Specificity is also important but biopsying an E is not such a mistake compared to the opposite situation. False negative rate reaches 26.5%, showing that, even in the hands of a specialist, there is a certain risk of choosing the wrong option with these tumors.

### 4.5. Management Algorithm Proposal

The last of our goals in the present study was the elaboration of a management algorithm (Figure 4), trying to integrate previous conclusions in the literature and our results. Several authors such as Weiner et al. [11–14] have reviewed the management of these tumors. Our proposal is focused on tumors of long bones of appendicular skeleton and includes our aggressiveness score in the distinction of these two entities (Table 2). From our point of view, there are two key decisions when studying these tumors: when we have to perform a complete imaging study including CT/MRI and bone scan and when we have to make a surgical decision regarding these patients under the suspicion of a LGC. The surgical technique preferred by the authors is an extensive intralesional resection associated with local adjuvant treatment (high-speed burr, phenolization, lavage with a high-pressure pulsatile system, and then packing the defect with cement). An additional internal fixation was indicated when needed (mainly distal femur) [15–23].

Those patients having a low aggressiveness cartilage tumor in long bones should be considered for further imaging studies if they have an inflammatory pain or pain with palpation is found. Even if many authors abandon the clinical signs as a reason to request more imaging studies, we think that clinical signs make the difference when a radiograph did not help with the diagnosis. Traditionally, CT scan, MRI scan, and Tc99 bone scan were included in the study. In our series, cortical involvement of any degree in both CT and MRI has shown significant relationship with those tumors that were finally considered as LGC in the biopsy. Moreover, MRI showed itself as the best tool to differentiate LGC from E according to cortical involvement. Geirnaardt et al. [7] concluded that a polilobular pattern, pop-corn calcification, absence of cortical involvement, and a nongeographical margin are indicative of an E. On the other hand, the presence of these features does not assure that the tumor is a LGC. In another paper, De Beuckeleer et al. [24] concluded that the use of contrast enhanced MRI can show features more typical of LGC such as arcs and rings enhancement, low signal septs, and lobulated tumors. Janzen et al. [9] found that peritumoral bone marrow contrast enhancement was more indicative of LGC in their series of 23 patients. Cortical involvement was not significant in their series. In our study, contrast was not employed because it is not a routine for cartilage tumors evaluation for our musculoskeletal radiologists. In addition, papers studying cartilage tumors have shorter series of patients compared to ours. Our proposal is to perform only an MRI and a Tc99 bone scan, leaving CT for those patients in which an MRI is not available (pacemakers, metal implants, etc.) or for doubtful cases, in which more information could be needed to complete the aggressiveness score.

With the results of the imaging, four situations are possible:

- With no cortical involvement in MRI:
  - No uptake or less than ASIC's in bone scan: clinical and radiological follow-up is our recommendation in these cases.
  - Uptake similar or higher than ASIC's. Doubts with these patients are logical with a clean MRI but strong metabolic activity. We recommend the use of the aggressiveness score and if 5 or more points are obtained, an intralesional resection with adjuvancies should be performed.
- With cortical involvement in MRI:
  - No uptake or uptake less than ASIC's in bone scan: doubts with these patients are logical with an MRI showing local damage but weak metabolic activity. We recommend the use of the aggressiveness score and if 5 or more points are obtained, an intralesional resection with adjuvancies should be performed.
  - Uptake similar or higher than ASIC's. In this situation in which signs of potential malignancy are present, an intralesional resection with adjuvancies is recommended.

**Figure 1:**
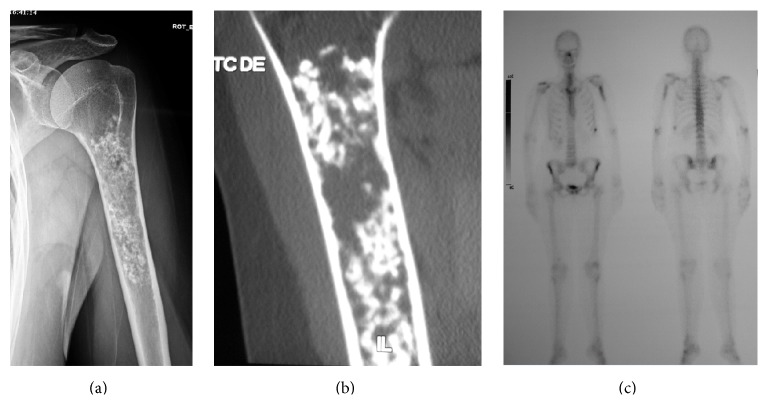
(a–c) A case of a big painless cartilage tumor with benign appearance in radiographs (a), endosteal scalloping in CT (b), and similar Tc99 uptake compared to ASIC. TA score was 5 and specimen analysis after intralesional resection with adjuvancies showed LGC.

**Figure 2:**
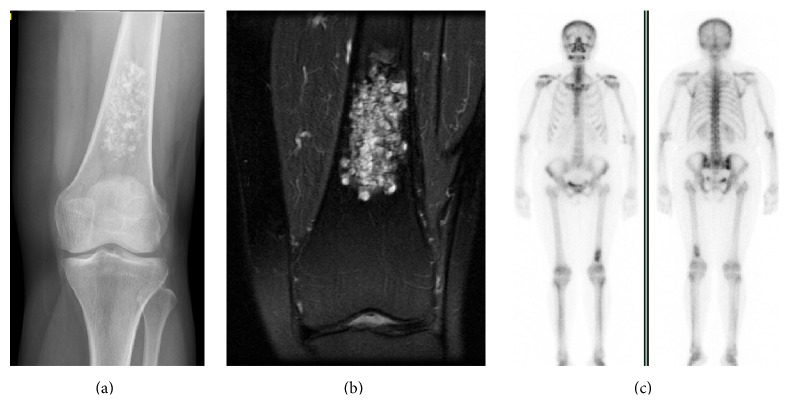
(a–c) Case of a tumor cartilage in distal femur (a) with cortical involvement in MRI (b) and increased Tc99 uptake compared to ASIC (c). TA was 5 and specimen showed LGC.

**Figure 3:**
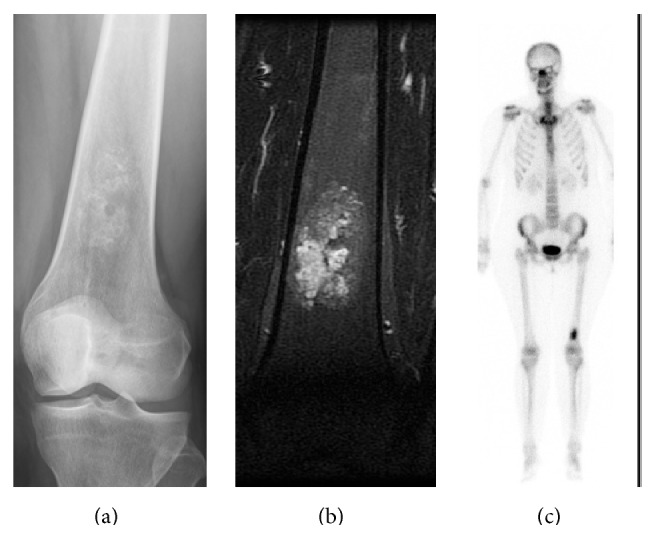
(a–c) Similar case of a tumor cartilage in distal femur (a) with cortical involvement in MRI (b) and increased Tc99 uptake compared to ASIC (c). TA was 5 and specimen showed enchondroma.

**Figure 4:**
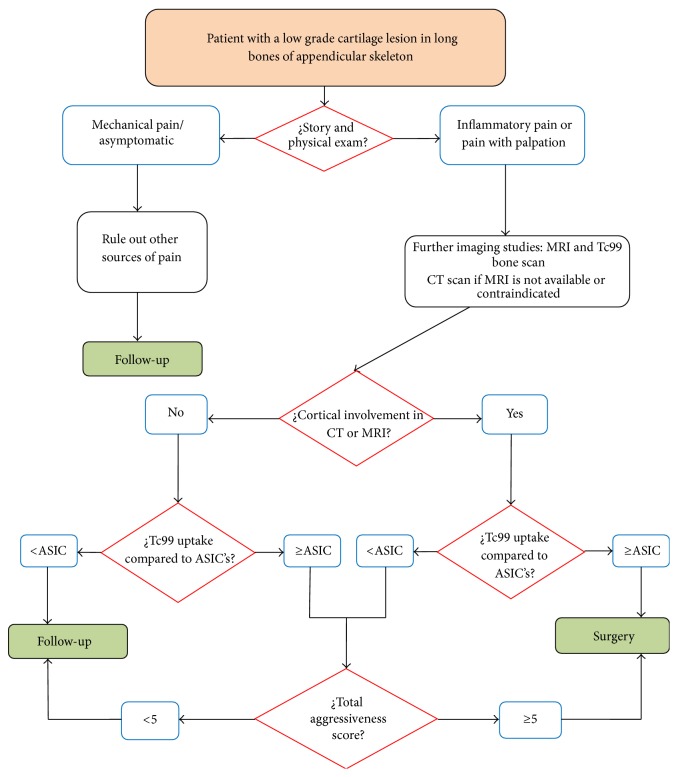
Management algorithm for low aggressiveness cartilage tumors in long bones of appendicular skeleton.

We think that the AS can also be used in those cases where not all the images are available as we mentioned above in the discussion; the final score is taken into account regardless of which categories the points are obtained in (see cases in Figures 1, 2, and 3).

## 5. Strengths and Weaknesses of the Study

The strong points of this study include the fact that we focused our analysis on tumors located in long bones where the diagnosis is more difficult. Moreover, we have excluded the chondrosarcomas of grade II according to the Evans classification. Prospective data collection and patients being interrogated and examined by the same surgeon, who is a widely experienced specialist in bone sarcomas, enabled us to have a uniform database to analyze. Weaknesses of the study include the fact that imaging reports have been made by different radiologists and nuclear medicine specialists, although the same surgeon evaluated all the images and the most borderline cases were presented at the weekly multidisciplinary meeting of sarcomas. Not all the imaging studies were available for every patient which is the reason why only 133 patients were finally included in the study. The fact that not all the patients have been biopsied is a major limitation of the study. Authors found it ethically unsuitable to perform a biopsy in a patient whose tumor has been an incidental finding, with no signs of aggressiveness and a very small size, just to confirm it is an E which does not require further treatment.

## 6. Conclusions

Distinction between E and LGC remains a challenge even for experts in bone sarcomas as shown in the results obtained when comparing an expert's first opinion and the final diagnosis in the biopsy. In our study, three features showed statistical relationship with LGC: pain on palpation, cortical involvement in CT/MRI, and Tc99 uptake similar to or higher than ASIC. These three features belong to the three categories in which we have divided our aggressiveness score. This score orientates the risk for a cartilage tumor in long bones of the appendicular skeleton to be a LGC rather than an E. This happens when the patient obtains 5 points or more. In view of all these facts, we have proposed a management algorithm which stresses the use of MRI, instead of CT, and Tc99 bone scan to complete the information provided by physical examination and plain radiographs. In case of strong suspicion of LGC, an intralesional resection with adjuvancies should be performed to obtain a suitable specimen for final diagnosis.

## Figures and Tables

**Table 1:** Compared features of solitary enchondroma and low grade chondrosarcoma.

Features	Solitary enchondroma	Low grade chondrosarcoma
Clinical	(i) Younger patients (ii) Pain is rare (iii) Typical in appendicular skeleton (iv) In general <5 cm	(i) Patients > 25 years (ii) Inflammatory pain (iii) Axial skeleton (iv) Bigger size

Radiological	(i) Intramedullary (ii) No periosteal reaction (iii) No endosteal scalloping (iv) No changes over time (v) No soft tissue mass	(i) Intramedullary (ii) Periostealreaction and microfractures (iii) Endosteal scalloping (iv) Loss of calcification. Increasing size (v) Soft tissue mass in some cases

Pathology	(i) Encasement pattern (ii) No endosteal scalloping (iii) Multinodular (iv) Surrounded by lamellar bone (v) No bone marrow infiltration	(i) Haversian system invasion (ii) Periosteal reaction and endosteal scalloping (iii) Single mass (iv) Occasional sites of necrosis and haemorrhage (v) Bone marrow invasion

**Table 2:** Aggressiveness score employed in our study.

Aggressiveness categories	Features (1 point per each of the following features)
Clinical aggressiveness CA	Presence of inflammatory pain Presence of pain with palpation

Radiological aggressiveness RA	Size bigger than 5 cm Metaphyseal location Loss of calcification (calcification lysis) over time Cortical involvement in CT or MRI Presence of a soft tissue mass in CT or MRI

Metabolic aggressiveness MA	Presence of Tc99 uptake in bone scan Uptake equal to or higher than anterosuperior iliac crest (ASIC)

Total aggressiveness TA	=CA + RA + MA

**Table 3:** Tumor location distributed by diagnosis.

Bone	Enchondroma	LGC
Femur	48.1%	37.3%
Humerus	36.7%	41.2 %
Fibula	6.3%	11.3%
Tibia	7.6%	9.8%
Ulna	1.3%	

**Table 4:** Bone site affected.

Bone site affected	Enchondroma	LGC
Proximal metaphysis	35.4%	40.4%
Distal metaphyso-epiphyseal	30.4%	19.6%

**Table 5:** Size measured on plain radiographs.

Size	Enchondroma	LGC
>5 cm	31%	40%

**Table 6:** CT cortical involvement.

Involvement depth	Enchondroma	LGC
1/3	61.1%	63.63%
2/3	22.2%	15.15%
3/3	16.6%	21.21%

**Table 7:** MRI cortical involvement.

Involvement depth	Enchondroma	LGC
1/3	62.5%	63.3%
2/3	**18.75%**	**13.33%**
3/3	18.75%	23.33%

**Table 8:** Bone scan uptake.

Technetium 99 bone scan	Enchondroma	LGC
=Uptake to anterosuperior iliac crest	44.1%	54.3%
<Uptake than anterosuperior iliac crest	35.3%	10.9%
>Uptake than anterosuperior iliac crest	20.6%	34.8%
